# The Return on Investment for the Prevention and Treatment of Childhood and Adolescent Overweight and Obesity in Beijing: A Modeling Study

**DOI:** 10.3390/nu16173006

**Published:** 2024-09-05

**Authors:** Zhenhui Li, Christina L. Meyer, Haiquan Xu, Angie Jackson-Morris, Man Zhang, Daphne Wu, Hairong He, Suying Chang, Guansheng Ma

**Affiliations:** 1 Department of Nutrition and Food Hygiene, School of Public Health, Peking University, Beijing 100191, China; 2 Laboratory of Toxicological Research and Risk Assessment for Food Safety, Peking University, Beijing 100191, China; 3 Center for Global Noncommunicable Diseases, RTI International, Durham, NC 12106, USA; 4 Institute of Food and Nutrition Development, Ministry of Agriculture and Rural Affairs, Beijing 100081, China; 5 School of Nursing, Peking University, Beijing 100191, China; 6 Beijing Center for Disease Control and Prevention, Beijing 100191, China; 7 Child Health Development Section, United Nations International Children’s Emergency Fund (UNICEF) Office for China, Beijing 100600, China

**Keywords:** childhood overweight and obesity, investment case, economic evaluation, health policy

## Abstract

**Background:** The increasing prevalence of child and adolescent overweight and obesity (CAOAO) in Beijing poses significant health and economic challenges. This study assesses the potential health and economic outcomes of implementing specific interventions to address CAOAO in Beijing. **Methods:** A deterministic Markov cohort model was used to estimate the impact of five interventions from 2025 to 2115: restrictions on unhealthy food marketing to children, mandatory front of package labeling (FOPL), family-based nutrition and exercise education, school-based nutritional health education, and nutritional counseling in primary healthcare. The model evaluated societal costs, healthcare savings, wages, and economic productivity in adulthood, calculating the return on investment (ROI) for each intervention and their combined effect. **Result:** Without intervention, Beijing is projected to experience a loss of 13.4 million disability-adjusted life years (DALYs) due to CAOAO. The health and economic impact of childhood obesity in Beijing is projected to be CNY 14.6 trillion (USD 2.1 trillion), or a lifetime loss of CNY 6.8 million (USD 0.96 million) per affected child, exceeding the sum of Beijing’s GDP from 2021 to 2023. Restrictions on unhealthy food marketing to children and nutrition counseling in primary healthcare are projected to yield the highest returns, with benefits accruing within one year of implementation. Owing to the substantial upfront costs, including personnel, materials, and training, school-based and family-based interventions require a longer time horizon to realize significant health and economic benefits. **Conclusions:** Effective management of CAOAO in Beijing demands a multifaceted approach. The combination of restrictions on unhealthy food marketing to children, mandatory front of package labeling, nutrition counseling in primary healthcare, school-based intervention, and family-based intervention presents the most substantial health and economic returns. This comprehensive strategy aligns with global best practices and addresses the unique challenges faced by Beijing in combating childhood obesity.

## 1. Introduction

Globally, child and adolescent overweight and obesity (CAOAO) incurs significant and economic impacts [1,2,3,4], and in China, these adverse effects have been calculated to be worth 2.19% of the 2019 global GDP [5]. China’s epidemiological shift has accompanied rapid economic growth, and overweight and obesity (OAO) prevalence among children and adolescents aged 0–19 years increased from 8.8% in 2000 to an estimated 37.9% in 2020—a 400% increase [6,7,8,9]. This surpasses the increase that other Western Pacific countries and upper-middle-income countries have undergone, affecting almost nearly two in every five Chinese children and adolescents [5]. If current trends persist, an estimated 60% of China’s youth could be affected by CAOAO by 2030 [6]. Without timely intervention, the projected loss between 2025 and 2105 could reach 3.3 billion disability-adjusted life years (DALYs) with an economic burden of 218 trillion CNY (31.6 trillion USD) [10].

The experience of CAOAO has been amplified in Beijing due to the high degree of urbanization and rapid economic development in China’s capital city. According to a 2022 study of OAO in Beijing, the OAO prevalence among 6–18 year olds in Beijing was 41.2% [11], exceeding the national average.

In response, the Chinese national government and Beijing city government have initiated several key policies, including ‘Healthy China 2030’ [12,13], ‘National Nutrition Plan 2017–2030’ [14], and ‘Guidelines for the Prevention and Control of Overweight and Obesity Among School Aged in Beijing’ [15]. These policies aimed to create a standardized framework for childhood obesity prevention, encompassing initiatives like establishing nutritionally healthy campuses and standardizing school meals nationwide.

Despite these proactive measures in Beijing, there remains a notable gap in the adoption of interventions that have been internationally recognized as effective [10]. Critical areas, such as restricting the marketing of unhealthy foods to children [16,17,18] and integrating nutrition advice into primary healthcare [19,20,21,22], have yet to be fully addressed. Moreover, health or economic evaluations of CAOAO interventions conducted to date have generally concentrated on school-based interventions [23,24,25,26,27]. This focus overlooks the wider potential economic and health benefits that could be realized from a more comprehensive, policy-driven approach to interventions to address CAOAO.

This study aimed to bridge this gap by estimating the health and economic impacts of CAOAO in Beijing. It aimed to identify and evaluate policies and interventions that could offer the greatest health and economic returns, taking account of Beijing’s unique challenges, such as the capital’s abundant availability of high-calorie foods, pervasive digital device usage fostering sedentary lifestyles, and urban infrastructure designed for convenience over physical activity. This study aligns with evolving urban public health policies under the “Healthy China 2030” and “Healthy Beijing 2030” initiatives, which emphasize prevention and integrating health in all policy.

## 2. Materials and Methods

This analysis was inspired by the ‘Return on Investment for the Prevention and Treatment of Childhood and Adolescent Overweight and Obesity in China’ study [10]. This study adapted the model and data analysis methodology used in the national study by prioritizing the use of local, Beijing-specific data and employing a different selection methodology to identify relevant interventions to assess.

Beijing was selected for this research due to its significantly higher childhood obesity rate compared to the national average, emphasizing the need for targeted intervention. As China’s capital, Beijing’s geographical, political, and cultural significance makes it a strategic location for developing interventions with potential nationwide impact. Its advanced health monitoring systems and extensive data collection provide a strong foundation for long-term research, while its concentration of medical, educational, and research institutions ensures professional support for implementing obesity interventions. Additionally, Beijing’s economic development and health-conscious population create an ideal environment for promoting healthy lifestyles and addressing childhood obesity.

### 2.1. Model Overview

The Microsoft Excel (Microsoft 365MSO, version number 2408)-based model used for the national investment case was used to analyze the health and economic impacts of various CAOAO interventions in Beijing. This model synthesized was integrated from two Markov cohort economic models: the ACE-Obesity model—focused on assessing future mortality impacts—and the EPOCH model—praised healthcare costs and efficacy of preventive strategies to address (CAOAO) [28,29].

The model adopted a societal cost perspective and forecast reductions in mortality and morbidity attributable to evidence-based prevention and treatment interventions. Additionally, this study estimated the interventions’ economic impact, including healthcare cost savings and their impacts on future wages and productivity.

The analytical time period was 2025–2115, focusing on a model cohort of children and adolescents living in Beijing aged 0–19 years in 2025. The model commenced in 2025 and ran through 2115 when the cohort met Beijing’s current maximum average life expectancy (90 years) [30]. The model operated under the premise that the full impact of the interventions would materialize one year following implementation [28,31].

#### 2.1.1. BMI, Overweight, and Obesity Prevalence

This study adopted globally recognized BMI standards to define overweight and obesity, with specific thresholds for adults, children aged 5–19, and under five year-olds from the World Health Organization (WHO) Growth References [4,10]. Projecting future mean BMI and OAO prevalence in China for the 0–19 age group in 2025, the analysis assumed constant mortality, morbidity, and risk factor trends. BMI was forecasted annually until age 19 and then in five-year intervals in adulthood to reflect the trends in BMI-OAO from childhood through adolescence to stability in adulthood. These projections, derived from multiple linear regression analysis of historical data from the NCD-Risk Factor Collaboration, informed the modeling of OAO prevalence across the life cycle [10,28,32].

#### 2.1.2. Health Consequences

Health impacts were quantified by calculating the number of years lost due to premature death (YLLs), years living with disabilities (YLDs), and the aggregate measure of these two, known as disability-adjusted life-years (DALYs), over the cohort’s lifespan. Historical mortality data from the United Nations Population Division [33] and disease-specific mortality data from 2000 to 2019 from the Global Burden of Disease study [31] were utilized to forecast future mortality rates and YLLs by cause, gender, and age under a nonintervention scenario. Under the intervention scenario, we assessed the decrease in YLLs and YLDs by applying the potential impact fraction (PIF) for each disease linked to obesity. PIF represents the relative change in mortality or morbidity due to a variation in exposure to a specific risk factor. The overall reduction in DALYs was determined by summing the reductions in YLLs and YLDs with health outcomes by gender [34].

#### 2.1.3. Economic Consequences

The analysis includes economic outcomes associated with CAOAO [10]: healthcare costs attributable to obesity during childhood and adulthood, and loss in wage income and productivity in adulthood due to CAOAO. To quantify the economic impact of life lost, we used Beijing’s 2022 gross domestic product (GDP) per capita as a multiplier, applying it to the years of life lost due to CAOAO-related mortality [5,35].

### 2.2. Intervention Selection for Beijing

CAOAO interventions were selected based upon the results of a systematic literature review and insights from a range of experts. The literature review drew from that conducted for the national investment case analysis, with an additional review of Beijing-specific literature. Details of the search methodology and findings are available in the national investment case and its Supplementary Materials documents [10]. Sources included Web of Science, PubMed, Google Scholar, and China Knowledge Infrastructure (CNKI) for relevant studies published from 1 January 2013 to 1 October 2023. The search incorporated a combination of keywords such as ‘China’, ‘Beijing’, ‘obesity’, ‘child’, ‘adolescent’, ‘investment case’, and ‘cost benefit’. The review provided a foundational understanding of the interventions that have been successful and economically viable in different contexts. World Health Organization (WHO) guidelines on child obesity were also examined [36,37,38,39,40,41,42].

To align interventions with the local context and enhance this study’s relevance and implement ability, we conducted interviews with ten experts from the Chinese government, Beijing municipal authorities, and academia, identified via the research team’s networks. The experts provided critical insights and recommendations regarding the most effective and suitable interventions for Beijing, based on the detailed criteria. These criteria included alignment with existing national policy guidelines, feasibility of implementation, public acceptance, and the government’s ability to absorb the intervention costs.

The experts offered critical insights and recommendations regarding the most effective and suitable interventions for Beijing (the interview guide is available in Supplementary Materials).

The five interventions to address CAOAO in Beijing were selected in three categories:

Policy interventions to create a healthier food environment (Policy):

1. *Restrictions on unhealthy food marketing to children and adolescents* limit advertising of unhealthy foods and beverages across all media channels for children and adolescents aged 2–18 years to reduce exposure to unhealthy dietary promotion.
2. *Mandatory Front of Package labeling* (*FOPL*): It will ensure that clear nutritional information is directly available to consumers, especially parents and caregivers, to support them in making healthier food choices for young people. A mandatory FOPL policy would replace China’s current voluntary FOPL system, which has seen limited adoption among food manufacturers.

Nutrition counseling

1. *Nutrition counseling in primary healthcare* would be administered by physicians to children and adolescents aged 0–19 who are affected by OAO. For younger children, the intervention includes parents or caregivers to equip them with guidance on implementing appropriate nutritional strategies at home.

School and family interventions

1. *Family-based intervention* focuses on primary-school-aged children and aims to link the school and family environments. The program uses educational workshops to promote healthy eating and physical activity in the home and family, with engagement and educational activities for families.
2. *School-based intervention* includes a structured nutrition education program in schools, regular assessments, school cafeteria menu improvements to ensure healthier food options, and implementation of the “Happy 10 Minutes” exercise sessions during the school day.

In addition to the five specific interventions, two comprehensive intervention packages were evaluated:

Package 1: a policy-focused approach that included restrictions on unhealthy food marketing to children/adolescents, alongside mandatory FOPL.

Package 2: a cross-sectoral approach, including restrictions on unhealthy food marketing to children/adolescents, FOPL, the school-based intervention, and nutrition counseling.

Table 1 sets out current intervention coverage for each intervention in Beijing, reflecting the status quo and the projected target coverage levels necessary for the five interventions to have an effective impact.

This study assumed baseline coverage of 0% for restricting marketing unhealthy foods to children, family-based intervention, and nutritional counseling in primary healthcare. Mandatory front of package labeling (FOPL) and school-based interventions started at 5% coverage, reflecting their current limited adoption. The FOPL intervention target coverage was 100%, as legislation for an FOPL intervention would have to be implemented at a national level by the National People’s Congress. The target for the nutrition counseling was 40%, in line with China’s 2017 National Basic Public Health Service Specification (Third Edition) and the Healthy China Action Plan (2019–2030). All other interventions had an 80% target coverage, adopting a gradual implementation approach.

### 2.3. Intervention Cost and Target Population

The cost estimation for interventions was calculated based on unit costs specific to Beijing, including historical city GDP, average wages, employment rates, Consumer Price Index (CPI), per capita healthcare spending, maximum expected lifespan, disease burden data, intervention effect sizes, and cost [31,44,45]. This was supplemented by global or other costs from other Chinese cities if local data were absent [23,43,46]. As the model cohort focused on the cohort of Beijing children and adolescents aged 0–18 in 2025, the model assumed the interventions were implemented from 2025 to 2044. This implementation timeframe was chosen to ensure that the intervention implementation period ended when all members of the cohort transitioned into adulthood, aligning with the period during which each child in the model cohort would no longer qualify for child- and adolescent-focused interventions.

The policy intervention category cost analysis encompassed planning, development, operations, management, and monitoring of policy interventions. However, it did not factor in potential economic impacts on GDP and tax revenue due to changes in unhealthy food sales, adhering to a societal perspective [10].

School-based intervention category costs included labor, materials, training, communication, and logistics related to implementing these programs in educational settings [44]. Additionally, family-based interventions involved costs to develop educational materials, organize training workshops, transportation, and communication [23].

### 2.4. Economic Outcome Indicator

We used return-on-investment (ROI) as the primary measure to evaluate the economic impact of interventions targeting CAOAO. ROI measures compare expected gains against costs and offer insights across different time horizons, including 10-, 30-, 50-years, or a lifetime horizon (90 years). This provides a nuanced understanding of immediate outcomes and enduring benefits, and so is helpful to inform policy and stakeholder decisions.

A standard discount rate of 3% per year was applied, aligned with global norms for health economic evaluations [35,47,48]. Costs and benefits were reported using 2022 CNY, providing a current value that reflects the long-term economic implications of the interventions.

The global GDP multiplier of 1.6 derived from life expectancy changes between 2000 and 2011 was applied to estimate the maximum economic value attributable to premature mortality, as per the Lancet Commission on Investing in Health’s recommendation [5].

Given the uncertainties in intervention measures, a sensitivity analysis that included adjusting GDP multipliers, intervention impacts, and discount rates was conducted. Detailed findings were provided in the Supplementary Materials.

## 3. Results

### 3.1. Cost on CAOAO

Under the status quo scenario where no new interventions are implemented, the cumulative health and economic burden of CAOAO in Beijing is profound. The model cohort from 2025 to 2115 is anticipated to experience a loss of 13.4 million disability-adjusted life years (DALYs)—a stark indicator of the health impact over the cohort’s lifetime (Table 2).

The total lifetime economic impact was projected to be CNY 14.6 trillion (USD 2.1 trillion) and exceed Beijing’s total GDP between the years 2021 and 2023 (Table 3).

Direct healthcare costs attributable to CAOAO during this period were estimated at approximately CNY 19.9 billion (USD 2.8 billion), with childhood and adulthood costs accounting for CNY 2.0 billion (USD 0.3 billion) and CNY 17.8 billion (USD 2.5 billion), respectively.

The indirect cost impact extends beyond direct healthcare expenditures. Indirect costs, including reduced productivity, wage losses due to diminished educational attainment, and the value of life lost, contribute substantially to the overall economic burden. Loss in lifetime wages and productivity losses due to absenteeism and presenteeism and educational underachievement result in CNY197.1 billion (USD 27.8 billion) and CNY 222.8 billion (USD 32.1 billion) in losses, respectively. The predominant losses are attributable to years of life lost, amounting to CNY 14.2 trillion (USD 1999.0 billion) (Table 3).

### 3.2. Cost Analysis of CAOAO Interventions in Beijing

Across the set of interventions assessed for Beijing, the cost spectrum varied significantly. Table 4 provided the cost of implementing the interventions between 2025 and 2044 in Beijing when the model cohort was eligible for child and adolescent interventions.

The lowest cost intervention was the policy restriction on unhealthy food marketing to children, at a total cost of CNY 4.5 million (USD 0.63 million). In contrast, the school-based intervention represents the highest financial commitment, amounting to CNY 3736.0 million (USD 526.0 million). Collectively, the total cost for implementing all proposed interventions—encompassing educational, policy, and healthcare initiatives—is estimated at CNY 3897.8 million (USD 548.81 million).

### 3.3. Health and Economic Benefits of Implementing New Interventions

Table 5 details the potential health benefits that could be achieved through implementing the interventions and the combined packages over the model cohort’s lifetime, quantified in terms of disability-adjusted life years (DALYs) averted.

**Figure 1:**
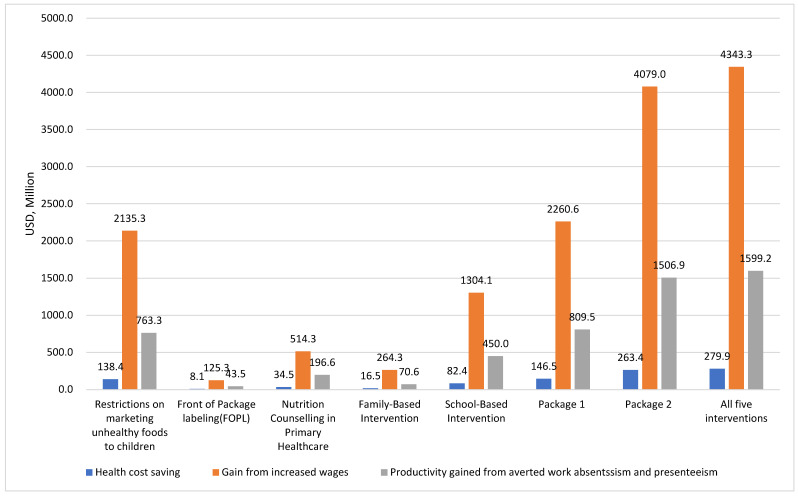
Total lifetime healthcare savings and gains in wages and productivity, 2025–2115 (USD, Millions).

Figure 1 provides an overview of the national economic gains, including lifetime healthcare savings and increases in wages and productivity. The collective implementation of all interventions is projected to reduce healthcare expenditures by CNY 2.1 billion (USD 279.9 million), increase wage gains by CNY 31.3 billion (USD 4343.3 million), and prevent production losses of CNY 11.5 billion (USD 1599.2 million).

Table 6 illustrates the benefits derived from each of the individual interventions and the substantial gains achievable from their collective implementation. When all five interventions are applied together, the model forecasts a total benefit of CNY 284.8 billion (USD 42.5 billion) from combined health improvements, healthcare cost savings, economic gains in productivity and wages, and economic value of life years gained.

### 3.4. ROI Analysis of Interventions in Beijing

The economic returns for all five interventions in Beijing progressively increase over time. Marketing restrictions and nutrition counseling in primary healthcare show immediate benefits, with their lifetime ROI increasing to 33,723.08 and 955.23, respectively (Table 7). The FOPL, school-based, and family-based interventions have more gradual positive economic returns, consistent with the gradual effects that improved education and awareness can have on behavior change over time, especially among children and adolescents. FOPL becomes economically beneficial after ten years, while School and Family Interventions take thirty years to yield positive ROIs. Notably, Package 1, focused solely on policy interventions, significantly outperforms the more comprehensive Package 2 in terms of ROI.

## 4. Discussion

In Beijing, the lifetime economic burden attributed to child and adolescent overweight and obesity (CAOAO) for the 2025 cohort of individuals aged 0–19, over a 90-year period, is projected to reach 14.6 trillion RMB (about 3.1 trillion USD), exceeding the combined value of Beijing’s GDP between 2021 and 2023. This significant impact underscores the urgent need for effective public health interventions.

The efficacy of various interventions and combinations in reducing the health and economic impacts of CAOAO markedly differed. The assumption that more interventions led to an increased ROI was not universal. While broader intervention strategies enhanced health outcomes—for instance, a combined package, including restrictions on unhealthy food marketing to children, mandatory front-of-package labeling, nutrition counseling, and school-based interventions significantly cut down disease burden—the increased implementation costs associated with adding more interventions paradoxically lowered ROI. Specifically, policy interventions like restrictions on unhealthy food marketing yielded the highest ROI, with every yuan invested generating a lifetime return of 33,723. However, although Package 2 had the greatest health benefits, it achieved a lower ROI of 75.96. This discrepancy, consistent with the national study’s findings [10], suggested that accumulating interventions, particularly school-based and health systems-focused interventions, could escalate costs, whereas policy-driven interventions maintained low long-term expenses.

Recent initiatives by China’s central and Beijing municipal governments to tackle CAOAO included diverse policies, actions, and educational media campaigns. Although the national and municipal CAOAO prevention strategies primarily rely on leadership and action by the education and health sectors, the findings of this study suggest that diversifying the range of policies and programs to address the systemic and environmental CAOAO risk factors would be key to slowing the current upward trends and mitigating the associated economic burden. At the same time, the different levels of health and economic returns on investment between the interventions and combined intervention packages demonstrate the government’s need to carefully assess and prioritize interventions, balancing financial constraints against the urgency of combating childhood obesity. This strategic selection is vital for aligning fiscal capacity with public health goals and ensuring efficient resource allocation to curb the CAOAO trend and its economic impact.

In addressing CAOAO, the Beijing municipal government can leverage its significant administrative influence to swiftly implement family-based and school-based interventions through educational and community channels, contingent upon strong municipal commitment. These interventions, while effective within the educational and social system, often require substantial investment and extended periods to realize returns. Conversely, interventions such as restrictions on unhealthy food marketing to children, mandatory front-of-package labeling (FOPL), and nutrition counseling in primary healthcare necessitate national legislative measures but are likely to yield swift and tangible benefits.

This distinction highlights the different roles and responsibilities of municipal and national governments in addressing CAOAO. National strategies focus on establishing legislative frameworks essential for effective obesity prevention, while municipal strategies prioritize the execution of these interventions to ensure all children and adolescents benefit. This necessitates a coordinated strategy that aligns local actions with national policies to enhance the effectiveness of public health interventions against CAOAO. Recommendations for each government level advocate for national legislative support and diligent local implementation to ensure comprehensive and effective health measures.

Similar to China’s national trends, the full impact of the interventions on Beijing’s child and adolescent OAO unfolded over a lifetime, causing initial delays in benefits. Interventions such as mandatory labeling family and school-based programs exhibited negative ROI within 10 years. Yet, significant returns emerged after 30 years for each intervention, underscoring the importance of long-term investment in combating the health and economic consequences of OAO in China.

The first analysis estimates the return on investment for multiple interventions against childhood obesity in Beijing, boasting significant strengths while acknowledging certain limitations. This study prioritizes locally relevant data, utilizing Beijing-specific information. Additionally, the selection of interventions and their baseline/target coverage parameters were meticulously chosen based on insights from stakeholder experts and supported by empirical data from studies conducted within China.

However, while Beijing-specific data were prioritized, supplementation with national and global data was necessary, particularly for intervention effect sizes [10,23,31,43,44,45]. The inclusion of these diverse data sources may introduce variability in data quality, highlighting the need for continuous refinement and improvement of data accuracy in future studies. Also, this approach assumes uniform effectiveness of interventions across diverse demographic segments within Beijing, potentially overlooking the varied impacts on different genders, regions, and socioeconomic groups. Additionally, the projections are based on the current trajectory of obesity interventions in Beijing without considering potential future public health measures. This assumption may not fully capture the evolving landscape of obesity management in the city.

## 5. Conclusions

This analysis estimates the economic benefits of five interventions targeting childhood and adolescent overweight and obesity in Beijing, China. By demonstrating the potential impact these interventions have on improving the health of children and economic returns to the city, the results provide an evidence base that can be used to strategically develop novel public health policies to address the obesity epidemic among the youth and strengthen existing ones. Policy-driven interventions often yield a higher return on investment, but the high costs and delayed return on investment for school-based interventions could present barriers to their implementation. As the first CAOAO investment case analysis was conducted at a municipal level, this analysis provides an example of how local stakeholders can develop evidence-based strategies to address CAOAO.

## Figures and Tables

**Table 1:** Baseline and target for the intervention in Beijing.

Intervention	Intervention Target Population	Beijing Baseline (%)	Modeled Target (%)
**Policy**			
Restrictions on unhealthy food marketing to children [43]	Children and adolescents aged 2–18 years	0	80
Mandatory Front of Package labeling (FOPL) [43]	All age label users	5	100
**Nutrition Counseling**			
Nutrition counseling in primary healthcare [43]	Children and adolescents affected by OAO aged 0–19 years	0	40
**School and Family Interventions**			
Family-based intervention [24]	School children aged 6–7 years	0	80
School-based intervention [25,44]	School children and adolescentsaged 6–17 years	5	80

**Table 2:** Health outcomes for the model cohort in the base case.

	YLL (Million)	YLD (Million)	DALYS (Million)
Male	5.8	1.5	7.3
Females	3.8	2.3	6.2
Total	9.6	3.8	13.4

**Table 3:** Direct and indirect costs attributable to childhood and adolescent overweight and obesity in Beijing, 2025–2115 years total (CNY/USD, Billion, 2022).

	Total Cost USD (Billions)	Total Cost CNY (Billions, 2022)	Average Lifetime Cost per Child with Obesity (USD)	Average Lifetime Cost per Child with Obesity (CNY, 2022)
**Direct healthcare costs**				
During childhood	0.3	2.0	296.2	2103.0
During adulthood	2.5	17.8	2661.7	18,897.8
Total direct healthcare costs	**2.8**	**19.9**	**2957.9**	**21,000.8**
**Indirect costs**				
Loss in lifetime wages	27.8	197.1	13,160.2	93,437.3
Productivity loss	32.1	228.2	2208.2	15,678.5
Mortality costs	1999.0	14,197.5	947,926.8	6,730,280.5
Total indirect costs	**2058.9**	**14,622.8**	**963,295.2**	**6,839,396.3**

**Table 4:** Cost of implementing child and adolescent overweight and obesity interventions, Beijing, 2025–2044 (USD/CNY, Million, 2022).

Intervention Cost	Beijing (Million, USD)	Beijing (Million, CNY, 2022)
**Policy**		
**1.** Restrictions on unhealthy food marketing to children	0.63	4.5
**2.** Mandatory Front of Package labeling (FOPL)	3.73	26.5
**Nutrition Counseling**		
**3.** Nutrition counseling in primary healthcare	5.55	39.5
**School and Family Interventions**		
**4.** Family-based intervention	12.87	91.4
**5.** School-based intervention	526	3736
**Package**		
Package 1 (Restrictions on unhealthy food marketing to children + FOPL)	4.36	31
Package 2 (Restrictions on unhealthy food marketing to children + FOPL + School-based intervention + Nutrition counseling in primary healthcare)	535.95	3806.4
All five interventions	548.81	3897.8

**Table 5:** Impact of interventions on DALY reductions during the lifetime of the model.

Intervention	Lifetime Reduction in DALYs
**Policy**	
**1.** Restrictions on unhealthy food marketing to children	146,629
**2.** Mandatory Front of Package labeling (FOPL)	8430
**Nutrition Counseling**	
**3.** Nutrition counseling in primary healthcare	34,917
**School and Family Interventions**	
**4.** Family-based intervention	8438
**5.** School-based intervention	83,062
**Package**	
Package 1 (Restrictions on unhealthy food marketing to children + FOPL)	155,436
Package 2 (Restrictions on unhealthy food marketing to children + FOPL + School-based intervention + Nutrition counseling in primary healthcare)	281,045
All five interventions	290,304

**Table 6:** Economic gains resulting from the five interventions, 2026–2115 (USD, millions).

Intervention	Healthcare Cost Savings (USD Millions)	Gains from Increased Wages (USD, Million)	Productivity Gained from Averted Work Absenteeism and Presenteeism (USD Millions)	Economic Value of Life Years Gained (YLLs only) (USD Millions)	Total Savings (USD Millions)
**Policy**					
**1.** Restrictions on unhealthy food marketing to children	138.4	2135.3	763.3	18,171.4	21,208.4
**2.** Mandatory Front of Package labeling (FOPL)	8.1	125.3	43.5	1040.0	1216.9
**Nutrition Counseling**					
**3.** Nutrition counseling in primary healthcare	34.5	514.3	196.6	4566.4	5311.8
**School and Family Interventions**					
**4.** Family-based intervention	16.5	264.3	70.6	720.3	1071.7
**5.** School-based intervention	82.4	1304.1	450.0	10,400.0	12,236.4
**Package**					
Package 1 (Restrictions on unhealthy food marketing to children + FOPL)	146.5	2260.6	809.5	19,268.9	22,485.5
Package 2 (Restrictions on unhealthy food marketing to children + FOPL + School-based intervention + Nutrition counseling in primary healthcare)	263.4	4079	1506.9	35,398.5	41,247.8
All five interventions	279.9	4343.3	1599.2	36,234.6	42,457.1

**Table 7:** Beijing return on investment (ROI) of selected child and adolescent obesity and obesity interventions over a 10-year, 30-year, 50-year, and lifetime time horizon.

Intervention	Return on Investment (ROI)			
	Over 10 Years	Over 30 Years	Over 50 Years	Over Lifetime
**Policy**				
**1.** Restrictions on unhealthy food marketing to children	287.37	2390.98	13,427.79	33,723.08
**2.** Mandatory Front of Package labeling (FOPL)	1.85	22.43	130.33	324.91
**Nutrition Counseling**				
**3.** Nutrition counseling in primary healthcare	8.55	80.08	438.08	955.23
**School and Family Interventions**				
**4.** Family-based intervention	−0.76	8.51	13.63	82.30
**5.** School-based intervention	−0.90	0.73	8.78	22.26
**Package**				
Package 1 (Restrictions on unhealthy food marketing to children + FOPL)	43.01	364.21	2049.67	5152.86
Package 2 (Restrictions on unhealthy food marketing to children + FOPL + School-based intervention + Nutrition counseling in primary healthcare)	−0.51	4.55	30.29	75.96
All five interventions	−0.51	4.66	29.61	76.36

## Data Availability

Data are contained within the article and Supplementary Materials document.

## Supplementary Materials

The following supporting information can be downloaded at: https://www.mdpi.com/article/10.3390/nu16173006/s1, Table S1: Overview of Interventions for Childhood and Adolescent Overweight and Obesity: Comparative Analysis by Type, Geographic Focus, and Language of Publication, Table S2: Cost Data for each intervention, Table S3: Baseline Level of Interventions in China and Effect Size to Reach Target Goals, Table S4: Intervention selection criteria, Tables S5–S9: National return on investment (ROI) of selected childhood obesity interventions in various sensitivity analysis scenarios.
