# Supplementary material for: The Return on Investment for the Prevention and Treatment of Childhood and Adolescent Overweight and Obesity in Beijing: A Modeling Study
Source: Nutrients. 2024 Sep 5;16(17):3006. doi: 10.3390/nu16173006 (PMC11396931; doi:10.3390/nu16173006)
Supplement: Supplementary file 1 [file nutrients-16-03006-s001.zip › nutrients-3191459-supplementary.pdf]

Supplementary materials

Supplementary materials 1

Systematic literature review for children and adolescents affected by obesity: Global, China and Beijing 2

Intervention unit cost details and intervention effect size 4

Intervention Selection Questions 7

Interview result summary 9

Intervention selection criteria 11

Sensitivity Analysis 11

Reference 15

Systematic literature review for children and adolescents affected by obesity: Global, China and Beijing

This review systematically collated evidence on various strategies effective in curbing or managing high body mass index (BMI), obesity, and overweight among the young population, spanning those at the societal level to clinical approaches and targeting familial contexts, prenatal, breastfeeding phases, and early childhood. Criteria for inclusion encompassed studies released from January 2010 onwards, documented in either English or Chinese. Research databases queried included PubMed, Web of Science for English publications, and CNKI along with Wanfang Data for Chinese-language studies. Systematic review software Rayyan facilitated the removal of duplicate entries. Subsequent phases involved detailed screenings of abstracts and full texts, applying specific inclusion and exclusion parameters. Studies merely identifying risk factors without proposing specific interventions were omitted. The search process is outlined in a flowchart available with our original research published by The Lancet journal. Following this methodology, we updated our initial search results, ensuring a thorough and current review of relevant studies.

Search Parameters for Interventions: The strategy encompassed terms relating to various intervention methodologies, encompassing prevention, management, marketing approaches, breastfeeding, social and mass media marketing, fiscal policies, physical activities, nutrition labeling, and modifications to the school food environment.

Cost Evaluation Parameters: Searches extended to keywords on cost-effectiveness, economic impact assessments, analyses of direct and indirect costs, savings, expenditures related to healthcare, and the economic aspects of public health.

Metrics for Intervention Efficacy: Emphasis was placed on metrics indicating changes in physical dimensions, including weight, BMI, waist circumference, body fat percentages through skinfold thickness, waist-to-height ratios, and bioelectrical measurements, to assess intervention outcomes.

Table S1: Overview of Interventions for Childhood and Adolescent Overweight and Obesity: Comparative Analysis by Type, Geographic Focus, and Language of Publication

Intervention type	Global interventions (English publications)	Interventions in China (English publications)	Global interventions (Chinese publications)	Interventions in China (Chinese publications)
**School**	36	6	9	10
**Family**	14	0	0	0
**Multiple components**	11	1	0	2
**Primary care**	10	0	1	1
**Taxation**	9	1	0	1
**Community**	6	0	0	2
**Residential activity camp**	5	0	1	1
**E-health**	5	0	1	1
**Physical activity**	4	1	1	2
**Bariatric surgery**	4	0	0	0
**Advertising / marketing**	3	0	0	0
**Childcare**	2	0	0	0
**Infant**	2	0	1	1
**Subsidy**	1	0	0	0
**Media**	1	0	0	0
**Hospital in-patient**	0	0	0	0
**Pharmacotherapy**	0	0	0	0
**Total**	**113**	**9**	**14**	**21**

Intervention unit cost details and intervention effect size

The examination of global and local nutritional guidelines, enriched by dialogue with nutrition experts, laid the foundation for selecting effective obesity prevention strategies for young populations in China. Sector-wide interviews illuminated the interventions most suited to China's unique context, as determined by a panel of pediatric obesity specialists. This selection process, rooted in WHO recommendations, strategically omits standalone school activity programs and front-of-package labeling due to their limited effectiveness and adoption in the Chinese landscape.

This analytical framework aims to underscore the social and economic advantages of bridging current intervention gaps, either by strengthening existing measures or pioneering new approaches. Leveraging evidence from national and international studies, the analysis assesses each intervention's impact on BMI and cost implications, prioritizing local data when available.

Forecasting from 2025 to 2044, the model applies intervention costs to a cohort to predict long-term outcomes. It adopts a gradual coverage increase strategy for interventions, aligning with health policy objectives and underscoring the necessity of phased implementation. A comprehensive summary of intervention impacts and financial estimates is meticulously compiled for accessibility.

See **Table S2 -S3**for intervention parameters, including effect size and unit costs.

Table S2: Cost Data for each interventions

Unit cost of 5 interventions

**Intervention**	Unit cost (cost per person per year)	Unit cost currency	Unit cost currency year	Intervention cost components
1.Family Base Interventions (Knowledge + Diet +Sport (Outside + Inside) +1year)**[1]**	10.01	United states	2015	Costs cover program setup, staff training, extra lessons, and materials like flyers and books.
2. School Based Interventions (Nutrition Education Intervention+ Happy 10+ 1 Year)**[2]**	26.80	United states	2010	Expenditures include staff education, additional teaching, and educational tools such as brochures and books.
3. Restrictions On Unhealthy Food Marketing To Children**[3]**	0.01	United states	2005	Financial planning entails costs for development, operations, administration, and monitoring.
4. FOPL **[3]**	0.05	United states	2005	Budgeting involves development, execution, administrative tasks, and program oversight expenses.
5. Nutrition Consulting**[3]**	0.47	United states	2010	Additional costs involve physician and staff time (25 minutes for 2.6 sessions), lab fees, healthcare training, and initial organization expenses.

Table S3: Baseline Level of Interventions in China and Effect Size to Reach Target Goals

Effect size of 5 interventions

**Intervention**	Effect size population	Effect size in terms of BMI reduction	Implementation target coverage	Implementation baseline coverage
1.Family Base Interventions (Knowledge + Diet +Sport (Outside + Inside) +1year) **[1]**	Children aged 6-7 (first year)	-0.13	80%	0%
2. School Based Interventions (Nutrition Education Intervention+ Happy 10+ 1 Year)**[2]**	Children aged 6-13.9	-0.29	80%	5%
3. Restrictions on Unhealthy Food Marketing To Children) **[3]**	Children of all ages	-0.41	80%	0%
4. FOPL**[3]**	All ages	-0.02	100%	5%
5. Nutrition Consulting**[3]**	Children aged 0-19 affected by overweight and obesity	-0.47	40%	0%

Intervention Selection Questions

Question

1. Please briefly describe your experience working with childhood and adolescent obesity and the specific interventions or policies that you have been a part of?

2. Thinking about the childhood and adolescent obesity interventions or policies that you have been involved with, or are aware of, can you describe some of the key successes? How were these evaluated? What factors do you think were most important in achieving those successes?

3. Thinking about the childhood and adolescent obesity interventions or policies that you have been involved with or are aware of, can you describe some of the key barriers that were encountered?

    - What strategies were employed to overcome these barriers?

4. Have any interventions or policies related to childhood and adolescent obesity not been successful, and if so, can you please explain why?

5. Beijing has implemented several policies designed to reduce obesity prevalence. Are there any changes to existing policies that you would recommend increasing their effectiveness?

6. Which policies or interventions do you believe could be the most cost-effective and feasible given the political and economic context in Beijing?

7. Generally, what capacity gaps or areas for strengthening should be considered when selecting policies that address Beijing's context?

8. What communication or dissemination strategies and messages are most likely to be successful for different audiences for the policies/interventions you identified above? For example, how might different strategies or policies be employed for:

   1) Policymakers
   2) Politicians
   3) The public
   4) Private industry?
      - Who do you see as the most important private sector actors and how do you think they are likely to respond to policy reforms aimed at reducing childhood and adolescent obesity?

9. Is there anything else you would like to share regarding childhood and adolescent obesity in Beijing and the selection of interventions that should be included in a childhood and adolescent obesity investment case model that has not been covered in these questions

Interview result summary

Drawing on the insights from 10 one-on-one interviews with experts in the field of childhood and adolescent obesity from the Chinese government, Beijing's municipal authorities, and academia, a comprehensive overview emerged, highlighting the complexities and potential pathways to addressing obesity in young populations. The discussions revolved around their experiences with various interventions and policies, successes and challenges faced, recommendations for enhancing existing policies, and suggestions for new, cost-effective measures suitable for Beijing's unique socio-political and economic landscape.

Q1: Expertise and Involvement

Interviewed experts, all of whom possess professorial ranks or higher and PhDs, shared over ten years of rich experience in nutrition and childhood obesity prevention. They have played key roles in formulating, implementing, and assessing various interventions and policies. Their expertise has significantly influenced the development of critical initiatives, including the "Childhood Obesity Prevention and Control Action Plan," Healthy School Campaigns, and the National Fitness Project, spanning school-based programs, public health initiatives, and regulatory measures on food marketing.

Q2: Successes and Evaluation

Key successes included improved nutritional awareness among children and parents, reduced consumption of sugary drinks, stronger policy and increased physical activity in schools. Success was often measured through BMI reductions, increased participation in physical activities, and healthier eating habits. Critical success factors highlighted were multi-sectoral collaboration, community involvement, strong political will, and continuous public education.

Q3： Barriers and Strategies

Common barriers encountered were resistance from the food and beverage industry, cultural preferences for certain foods, and budget constraints. Strategies to overcome these barriers included stakeholder engagement, policy advocacy, public-private partnerships, and targeted financial investments.

Q4： Less Successful Interventions

Some interventions did not yield the expected results due to lack of enforcement, inadequate funding, or insufficient public buy-in. Experts emphasized the importance of robust implementation mechanisms and ongoing evaluation to adapt strategies as needed.

Q5: Cost-Effective and Feasible Policies

Experts highlighted the superiority of holistic approaches for cost-effectiveness, advocating for a blend of policy and action. This includes coupling restrictions on unhealthy food promotions targeted at children with in-school nutritional and health education. Such integrated strategies utilize the synergy between regulatory measures and educational initiatives to maximize impact.

Q6: Policy Recommendations for Beijing:

Policy Recommendations for Beijing: Experts emphasized the need to refine existing policies to maximize their effectiveness, specifically advocating for the rigorous implementation of the five key measures discussed in our study: Family-Based Interventions, School-Based Interventions, Restrictions on Unhealthy Food Marketing to Children, Front of Package Labeling (FOPL), and Nutrition Consulting. They recommended tighter controls on advertising unhealthy foods to children, increased investment in public health education that supports family and school interventions and improved urban planning to promote physical activity. These enhancements are aimed at creating a holistic and supportive environment for healthier lifestyle choices among children and adolescents in Beijing.

Q7: Capacity Gaps and Strengthening

Identified capacity gaps included insufficient training for healthcare providers on obesity management, lack of public awareness, and inadequate infrastructure for physical activities. Strengthening these areas is crucial for the effective implementation of obesity interventions.

Q8: Communication Strategies

Tailored communication strategies were deemed essential for engaging different stakeholders. For policymakers and politicians, evidence-based advocacy and highlighting the economic rationale for obesity prevention were recommended. Public campaigns should focus on easy-to-understand messages about healthy lifestyle choices, while engagement with the private sector should emphasize corporate social responsibility and potential for positive brand association.

Q9: Private Sector Engagement

The food industry and media were identified as key private sector actors. Experts believed that policy reforms, coupled with incentives for healthier product offerings and responsible advertising, could encourage positive responses from these sectors.

Q10: Additional Insights

Experts stressed the importance of a holistic approach that addresses the socio-economic determinants of health, suggesting that interventions shouldn’t only focus on behavior change but also on creating supportive environments.

Intervention selection criteria

The intervention selection criteria reflects that used in the national China investment case study[4]

Table S4: Intervention selection criteria

Criteria	Description
Impact	• Demonstrated impact on BMI reduction, with specifics on the magnitude of the effect.
Feasibility	• Insights from key informant interviews suggesting the practicability of implementing such interventions in Beijing.<br>• Instances of comparable interventions that have been implemented in China
Low cost per disability adjusted life years (DALY) averted	• The criterion for classifying an intervention as low-cost is if averting one DALY costs less than three times Beijing's per capita GDP.<br>• An intervention is deemed extremely cost-effective if averting a DALY cost less than Beijing's per capita GDP
Expert recommendation	• Support and advice derived from interviews with key stakeholders.

Sensitivity Analysis

In our sensitivity analysis, we assessed the stability of our findings against pivotal assumptions, focusing on GDP multipliers and the impact of a higher discount rate to determine the economic value of interventions against childhood and adolescent overweight and obesity.

Our initial approach utilized a global GDP multiplier of 1.6, integrating changes in GDP per capita with adjustments in mortality and life expectancy. To ensure a comprehensive analysis, we examined the effects of employing both a conservative GDP multiplier of 1 and a region-specific East Asia and Pacific GDP multiplier of 2.2. The latter aimed to offer an estimation reflective of the East Asia and Pacific economic context, potentially providing a more precise evaluation of the interventions' economic benefits within this region. The inclusion of a neutral multiplier of 1 allowed us to consider a less optimistic scenario, enhancing the relevance of our analysis across various economic conditions.

Furthermore, we conducted a sensitivity analysis with a higher discount rate of 5%,

recognizing the significance of immediate benefits in economic evaluations, particularly in low and middle-income countries. This adjustment led to a reduction in each intervention's ROI, highlighting how ROI estimates are sensitive to changes in the discount rate. Using a higher discount rate decreases each intervention's ROI.

To tackle uncertainties about the collective impact of interventions, we examined the ROI through two distinct scenarios:

1. Adjusting all interventions to achieve 100% target coverage, and alternatively;
2. Reducing each intervention's target coverage to half of its current values.

Table S5-S9: National return on investment (ROI) of selected childhood obesity interventions in various sensitivity analysis scenarios

Table S5

	**ROI by valuing one life-year as GDP per capita times global GDP multiplier (1.0) obtained from the Lancet Commission on Investing in Health (Jamison et al., 2013)**			
	Over 10 years	Over 30 years	Over 50 years	Over lifetime
**Restrictions on unhealthy food marketing to children**	287.27	2363.1	14125.78	35728.25
**FOPL**	1.85	22.14	137.14	344.24
**Nutrition Consulting in Primary Healthcare**	8.55	79.96	461.85	1012.02
**Family-Based Intervention**	-0.76	8.31	-5.11	86.23
**School-Based Intervention**	-0.9	0.71	9.29	23.63
**Package 1 (Restrictions on unhealthy food marketing to children + FOPL)**	42.99	359.96	2156.28	5459.35
**Package 2 (Restrictions on unhealthy food marketing to children + FOPL+ School-Based Intervention+ Nutrition Consulting in Primary Healthcare)**	-0.51	4.5	31.94	80.54
**All five interventions**	-0.51	4.61	31.19	80.94

Table S6

	**ROI by valuing one life-year as GDP per capita times regional GDP multiplier for East Asia and Pacific (2.2) obtained from the Lancet Commission on Investing in Health (Jamison et al., 2013)**			
	Over 10 years	Over 30 years	Over 50 years	Over lifetime
**Restrictions on unhealthy food marketing to children**	327.58	1872.38	26986.85	72808.39
**FOPL**	2.25	17.12	262.45	701.65
**Nutrition Consulting in Primary Healthcare**	9.97	81.76	904.6	2066.62
**Family-Based Intervention**	-0.76	4.31	-33.46	158.12
**School-Based Intervention**	-0.88	0.43	18.67	49.01

**Package 1 (Restrictions on unhealthy food marketing to children + FOPL)**	49.14	285.21	4120.7	11127.04
**Package 2 (Restrictions on unhealthy food marketing to children + FOPL+ School-Based Intervention+ Nutrition Consulting in Primary Healthcare)**	-0.44	3.66	62.29	165.3
**All five interventions**	-0.45	3.71	60.32	165.67

Table S7

	**ROI using an annual discounting rate of 5%**			
	Over 10 years	Over 30 years	Over 50 years	Over lifetime
**Restrictions on unhealthy food marketing to children**	272.58	1580.2	16869.47	47944.11
**FOPL**	1.71	14.4	163.77	461.71
**Nutrition Consulting in Primary Healthcare**	8.18	62.48	570.16	1378.68
**Family-Based Intervention**	-0.77	3.68	-15.58	102.29
**School-Based Intervention**	-0.9	0.14	10.96	31.01
**Package 1 (Restrictions on unhealthy food marketing to children + FOPL)**	40.75	240.49	2575.44	7326.95
**Package 2 (Restrictions on unhealthy food marketing to children + FOPL+ School-Based Intervention+ Nutrition Consulting in Primary Healthcare)**	-0.53	2.72	37.65	106.15
**All five interventions**	-0.54	2.76	36.51	106.37

Table S8

	**Increase the intervention impact (All Interventions Targeted increase to 100% Coverage)**			
	Over 10 years	Over 30 years	Over 50 years	Over lifetime
**Restrictions on unhealthy food marketing to children**	307.42	2123.75	20629.58	54606.09
**FOPL**	2.05	19.63	199.79	522.94
**Nutrition Consulting in Primary Healthcare**	9.26	81.25	686.77	1558.55
**Family-Based Intervention**	-0.76	6.4	19.01	122.6
**School-Based Intervention**	-0.89	0.57	14.02	36.5
**Package 1 (Restrictions on unhealthy food marketing to children + FOPL)**	55.16	386.1	3758.73	9955.34
**Package 2 (Restrictions on unhealthy food marketing to children + FOPL+ School-Based Intervention+ Nutrition Consulting in Primary Healthcare)**	-0.4	4.88	53.96	140.4
**All five interventions**	-0.4	4.93	52.5	140.43

Table S9

	Decrease the intervention impact (All Interventions Effect Sizes Reduced by 50% from Original Effect Sizes Values)			
	Over 10 years	Over 30 years	Over 50 years	Over lifetime
**Restrictions on unhealthy food marketing to children**	307.42	2105.86	20445.75	53808.18
**FOPL**	2.05	19.63	199.79	522.94
**Nutrition Consulting in Primary Healthcare**	9.26	80.86	682.82	1535.06
**Family-Based Intervention**	-0.76	6.27	-19.36	121.79
**School-Based Intervention**	-0.89	0.56	13.9	36.01
**Package 1 (Restrictions on unhealthy food marketing to children + FOPL)**	57.57	399.25	3883.61	10224.08
**Package 2 (Restrictions on unhealthy food marketing to children + FOPL+ School-Based Intervention+ Nutrition Consulting in Primary Healthcare)**	-0.5	3.94	43.64	117.04
**All five interventions**	307.42	2105.86	20445.75	53808.18

Reference

1. Zanganeh, M.; Adab, P.; Li, B.; Pallan, M.; Liu, W.J.; Hemming, K.; Lin, R.; Liu, W.; Martin, J.; Cheng, K.K.; et al. Cost-Effectiveness of a School-and Family-Based Childhood Obesity Prevention Programme in China: The "CHIRPY DRAGON" Cluster-Randomised Controlled Trial. *Int J Public Health* **2021**, *66*, 1604025, doi:10.3389/ijph.2021.1604025.
2. Meng, L.; Xu, H.; Liu, A.; Van Raaij, J.; Bemelmans, W.; Hu, X.; Zhang, Q.; Du, S.; Fang, H.; Ma, J.; et al. The Costs and Cost-Effectiveness of a School-Based Comprehensive Intervention Study on Childhood Obesity in China. *PLoS ONE* **2013**, *8*, e77971, doi:10.1371/journal.pone.0077971.
3. Cecchini, M.; Sassi, F.; Lauer, J.A.; Lee, Y.Y.; Guajardo-Barron, V.; Chisholm, D. Tackling of Unhealthy Diets, Physical Inactivity, and Obesity: Health Effects and Cost-Effectiveness. *The Lancet* **2010**, *376*, 1775–1784, doi:10.1016/S0140-6736(10)61514-0.
4. Ma, G.; Meyer, C.L.; Jackson-Morris, A.; Chang, S.; Narayan, A.; Zhang, M.; Wu, D.; Wang, Y.; Yang, Z.; Wang, H.; et al. The Return on Investment for the Prevention and Treatment of Childhood and Adolescent Overweight and Obesity in China: A Modelling Study. *The Lancet Regional Health – Western Pacific* **2023**, *0*, doi:10.1016/j.lanwpc.2023.100977.
